# Altered brain-wide auditory networks in a zebrafish model of fragile X syndrome

**DOI:** 10.1186/s12915-020-00857-6

**Published:** 2020-09-16

**Authors:** Lena Constantin, Rebecca E. Poulsen, Leandro A. Scholz, Itia A. Favre-Bulle, Michael A. Taylor, Biao Sun, Geoffrey J. Goodhill, Gilles C. Vanwalleghem, Ethan K. Scott

**Affiliations:** 1 Queensland Brain Institute, The University of Queensland, St Lucia, Brisbane, QLD 4072 Australia; 2 School of Mathematics and Physics, The University of Queensland, Brisbane, 4072 Australia; 3 Australian Institute for Bioengineering and Nanotechnology, The University of Queensland, Brisbane, QLD 4072 Australia

**Keywords:** Fragile X syndrome, Autism spectrum disorder, Brain/physiopathology, Auditory perception, Sensory systems, CGaMP, Calcium imaging, Zebrafish, Light-sheet microscopy, Graph theory

## Abstract

**Background:**

Loss or disrupted expression of the *FMR1* gene causes fragile X syndrome (FXS), the most common monogenetic form of autism in humans. Although disruptions in sensory processing are core traits of FXS and autism, the neural underpinnings of these phenotypes are poorly understood. Using calcium imaging to record from the entire brain at cellular resolution, we investigated neuronal responses to visual and auditory stimuli in larval zebrafish, using *fmr1* mutants to model FXS. The purpose of this study was to model the alterations of sensory networks, brain-wide and at cellular resolution, that underlie the sensory aspects of FXS and autism.

**Results:**

Combining functional analyses with the neurons’ anatomical positions, we found that *fmr1*^*−/−*^ animals have normal responses to visual motion. However, there were several alterations in the auditory processing of *fmr1*^*−/−*^ animals. Auditory responses were more plentiful in hindbrain structures and in the thalamus. The thalamus, torus semicircularis, and tegmentum had clusters of neurons that responded more strongly to auditory stimuli in *fmr1*^*−/−*^ animals. Functional connectivity networks showed more inter-regional connectivity at lower sound intensities (a − 3 to − 6 dB shift) in *fmr1*^*−/−*^ larvae compared to wild type. Finally, the decoding capacities of specific components of the ascending auditory pathway were altered: the octavolateralis nucleus within the hindbrain had significantly stronger decoding of auditory amplitude while the telencephalon had weaker decoding in *fmr1*^*−/−*^ mutants.

**Conclusions:**

We demonstrated that *fmr1*^*−/−*^ larvae are hypersensitive to sound, with a 3–6 dB shift in sensitivity, and identified four sub-cortical brain regions with more plentiful responses and/or greater response strengths to auditory stimuli. We also constructed an experimentally supported model of how auditory information may be processed brain-wide in *fmr1*^*−/−*^ larvae. Our model suggests that the early ascending auditory pathway transmits more auditory information, with less filtering and modulation, in this model of FXS.

## Background

Fragile X syndrome is one of the most common causes of inherited intellectual disability in humans, with half of males and one fifth of females with FXS also meeting the diagnostic criteria for autism spectrum disorder (ASD) [1]. In most cases, FXS is caused by dominant X-linked mutations that suppress the expression of the fragile X mental retardation protein [2]. The vast majority of the mutations are caused by the expansion of a CGG-trinucleotide repeat within the 5′ untranslated region of the *Fragile X mental retardation 1 (FMR1)* gene.

One of the first clinical features of ASD are atypical responses to or interests in sensory stimuli [3]. These changes in sensory function likely result from the diminished ability to filter sensory information [4]. FXS is also associated with unusual responses to a variety of sensory stimuli. For example, individuals with FXS display impaired visual-motor functions that are inversely correlated to the expansion number of the trinucleotide repeat mutation in the *FMR1* gene [5]. These visual impairments are not generalized but appear to be selective to and pervasive in the magnocellular visual processing stream [6–8], an anatomically and functionally segregated visual pathway that controls motion perception. Furthermore, children and adults with FXS have abnormally large evoked electrophysiological responses to sound [9–11], which combined with human clinical and behavioral studies, point towards auditory hypersensitivity. Evidence is emerging that the subcortical auditory system underlies auditory sensitivities in FXS and ASD [12, 13], but the fine-scale circuit-level causes of these sensory phenotypes remain mysterious because of the technical challenges associated with observing functional sensory networks at cellular resolution. As a result, questions such as how localized changes in neural activity are integrated at the scale of entire sensory networks [14] are only beginning to be explored.

A leading theory for the neurobiological basis of FXS and ASD is that altered connectivity is responsible for behavioral and sensory phenotypes. However, functional magnetic resonance imaging studies report conflicting findings. For example, a reduction or loss of functional and structural connectivity has been widely documented in autism, while more recent studies suggest hyper-connectivity [15, 16]. Inconsistencies in the empirical evidence for hypo- or hyper-connectivity arise from a combination of factors including the heterogeneity and comorbidity found in FXS and ASD; the more frequent head movements in clinical populations that led to spurious and biased intensity-changes in signals [17]; greater inter-subject variability, or idiosyncratic distortions, in the connectivity patterns of ASD patients [18]; and differences in data analysis strategies that have profound downstream effects on interpretations [19]. Developmental age also dramatically affects brain connectivity. In ASD, pre-adolescent children consistently show global hyper-connectivity [20–22] whereas adolescents generally display hypo-connectivity [23, 24]. Functional studies on children with FXS are yet to be performed, but the neocortex of still-developing *Fmr1* knockout mice display altered synchrony [25, 26], hyper-connectivity [27, 28], and hyperexcitability [29, 30] that normalize in adulthood. Thus, early hyper-connectivity may give rise to hypo-connectivity across development in FXS, as occurs in ASD.

Discrepancies in the literature regarding hypo- or hyper-connectivity in FXS and ASD provide a powerful incentive to observe and model neural circuits in vivo during sensory processing in genetic models of FXS and ASD. Recent advances in microscopy [31], genetically encoded calcium sensors [32], and data processing [33] have made it possible to record and interpret neuronal activity at cellular resolution across large populations of neurons within the zebrafish brain [34], thus enabling brain-wide network-level studies of sensory processing to be performed in normal and disease contexts in zebrafish. We have performed whole-brain calcium imaging of visual and auditory sensory processing in wild-type (WT) zebrafish larvae and larvae with a mutation in the *fmr1* gene [2]. The objectives of this study were to observe how brain-wide neural networks in WT and *fmr1*^*−/−*^ mutant zebrafish process visual and auditory information during a critical developmental period, thus providing insights into the network-level causes of the mutant animals’ sensory processing phenotypes.

## Results

**Fig. 1:**
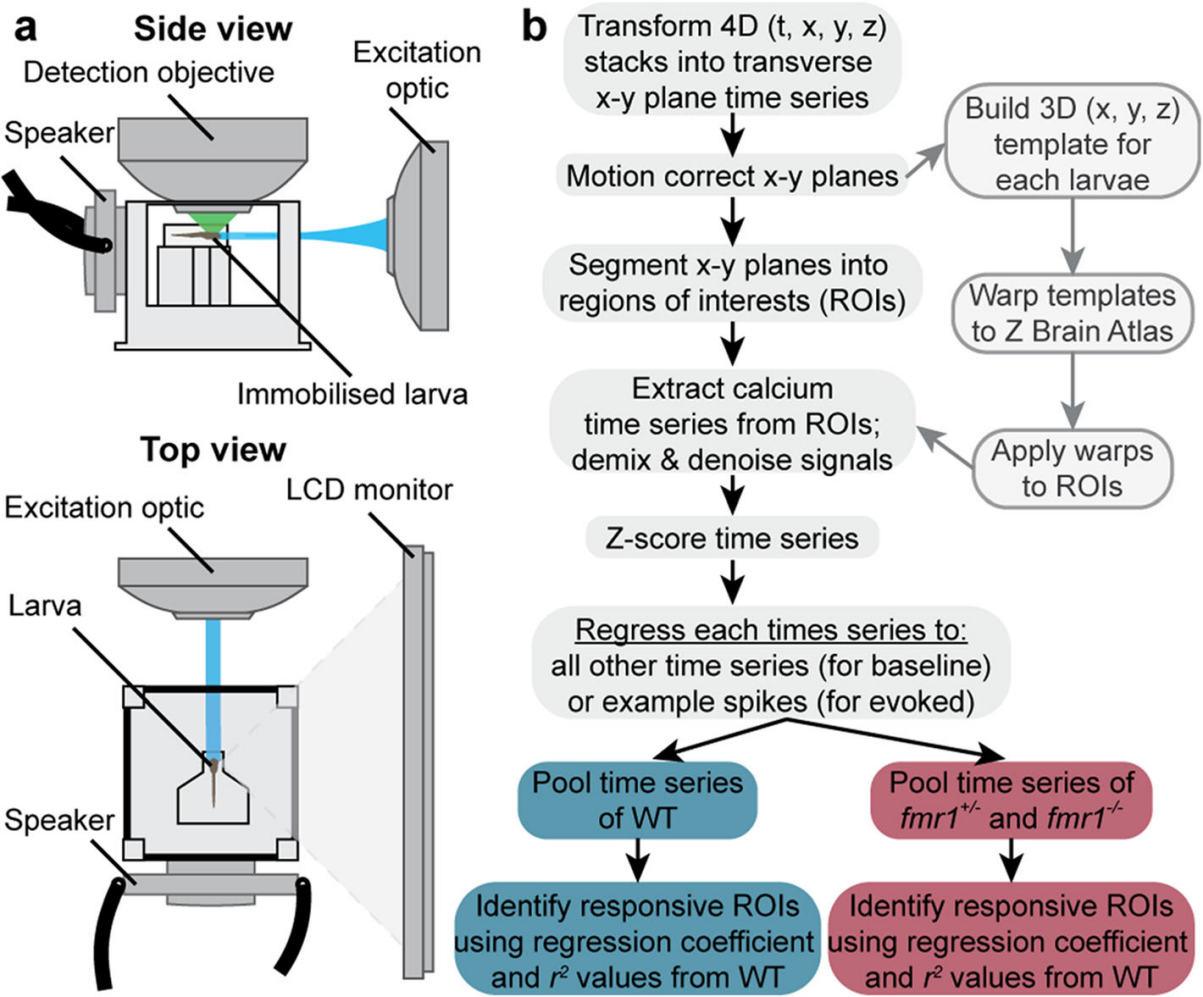
Imaging setup and neuroinformatic workflow. Schematic of the imaging setup (**a**) used to observe brain activity of zebrafish larvae. Summary of the neuroinformatic workflow (**b**) used to segment the images into ROIs, detect the activity, and then spatially register the response of each ROI to a reference brain

**Fig. 2:**
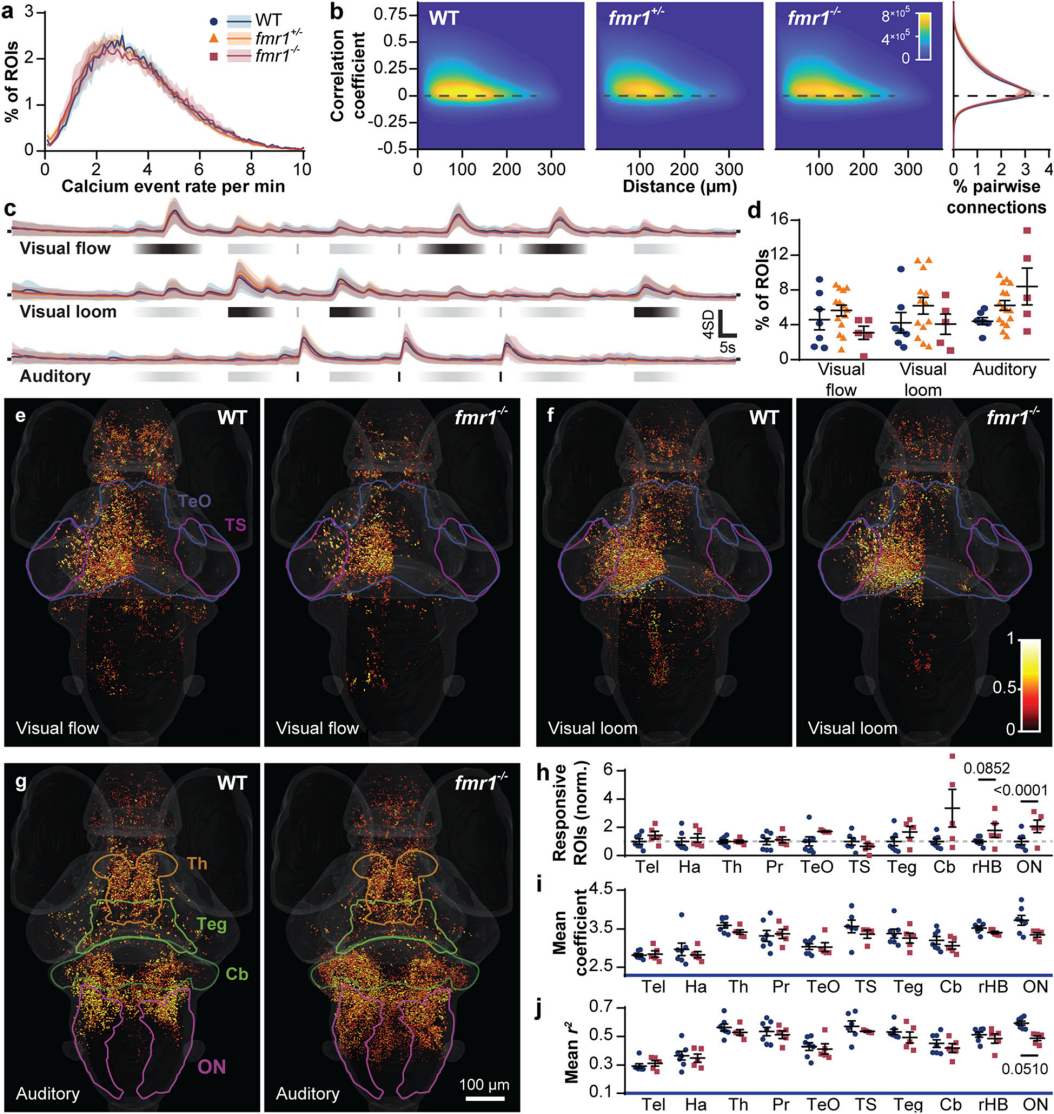
Brain-wide baseline and sensory-evoked neuronal activity. Distribution of brain-wide calcium event rates (**a**) in WT, *fmr1*^*+/−*^, and *fmr1*^*−/−*^ larvae at baseline (mean ± s.e.m.). Heat map (**b**) of the correlation coefficient between pairs of ROIs as a function of their Euclidean distance, with the distribution of all coefficients (right) showing no marked differences between genotypes. *Z*-scored activity traces (**c**) of all ROIs responding to stimuli for each genotype (mean ± SD). Percent of ROIs (**d**) in the whole brain responding to each stimulus (mean ± s.e.m). Brain-wide responses to visual flow (**e**), visual loom (**f**), and auditory (**g**) stimuli (all panels equivalent to five larvae), where spot color represents response strength (regression coefficient) and spot diameter depicts coefficient of determination (*r*^*2*^ value). Ratio of ROIs (**h**), mean regression coefficients (**i**), and *r*^*2*^ values (**j**) in WT versus *fmr1*^*−/−*^ responding to the auditory stimulus across various brain regions (mean ± s.e.m.). ON, octavolateralis nucleus; rHB, remaining hindbrain (without the cerebellum (Cb) and ON); Teg, tegmentum; TS, torus semicircularis; TeO, optic tectum; Pr, pretectum; Th, thalamus; Ha, habenulae; Tel, telencephalon. *P* values ≤ 0.1 are shown

Using GCaMP6s and GCaMP6f [32], combined with light-sheet microscopy [35], we performed volumetric imaging of both baseline and stimulus-evoked neuronal calcium activity at 2–4 Hz across the brains of 6 days post fertilization (dpf) zebrafish larvae (Fig. 1a). We subsequently segmented regions of interest (ROIs), generally corresponding to individual neurons, and extracted the fluorescence traces from each ROI (Fig. 1b). We first measured baseline neuronal activity in WT, *fmr1*^*+/−*^, and *fmr1*^*−/−*^ larvae, and found similar numbers of calcium events among the genotypes (Fig. 2a, Additional file 1). To determine whether correlations among active neurons had increased, as occurs in the cortex of adolescent *Fmr1*^*−/−*^ mice [25], we calculated the correlation coefficients between all ROIs for each larva. Again, there were no significant differences in mean correlations (Fig. 2b). These results suggest that *fmr1*^*−/−*^ animals have roughly normal baseline neuronal activity and activity correlations at 6 dpf.

Humans with FXS have deficits in visual motion detection [6–8] and hypersensitivity to auditory stimuli [10, 11]. To judge whether *fmr1*^*−/−*^ larvae have similar sensory phenotypes, we presented two visual stimuli (moving lines that provide visual flow and a looming disk) and one auditory stimulus (white noise at 84 decibels (dB)) to larvae while performing calcium imaging. To identify responsive ROIs, we used multivariate linear regressions to compare each *z*-scored calcium trace to regressors for the three stimuli. The regression coefficient provided a readout of response strength, while its *r*^*2*^ specified the proportion of variability that could be explained by the regression model. The *r*^*2*^ values are impacted by correlations between repeated stimuli (a measure of consistency) and by activity during stimulus-free intervals (a measure of noisiness). To tease apart what *r*^*2*^ represented in our datasets, we graphed the frequencies of the mean correlation between repeated stimuli across genotypes (a measure of consistency) and found no significant differences across stimuli between genotypes (Additional file 2). We interpreted this to mean that the differences in auditory sensitivity observed between genotypes were not the result of changes to the consistency of the responses and hence must have stemmed from genotype-specific alterations in noisiness. We calculated the mean *z*-scored fluorescent trace of responsive ROIs for each genotype to estimate the number and quality of responses across the whole brain (Fig. 2c) and the proportion of responsive ROIs per larva (Fig. 2d). These analyses showed similar activity profiles for all stimuli and similar proportions of visually responsive ROIs, but with a trend towards more auditory responsive ROIs in *fmr1*^*−/−*^ brains.

To explore whether the *fmr1* mutation altered responses in different brain regions, we mapped each responsive ROI back to its anatomical position within a reference brain. This allowed for the quantification of ROI number, response strength, and *r*^*2*^ values for different brain regions. We partitioned the brain into ten regions that together constituted 72–83% of the total ROIs detected in each fish. These regions were the cerebellum, habenulae, octavolateralis nucleus, pretectum, remaining hindbrain (without the cerebellum and octavolateralis nucleus), optic tectum, tegmentum, telencephalon, thalamus, and torus semicircularis. We note that two other brain regions of potential interest, the hypothalamus and tuberculum, were too ventral to be segmented reliably within our images and were therefore not analyzed further.

Across the brain, the distributions and strengths of the responses in our ten regions of interest were broadly similar between WT and *fmr1*^*−/−*^ animals for visual flow (Fig. 2e; Additional file 3) and visual loom (Fig. 2f; Additional file 3), and these parameters were quantitatively similar across all ten brain regions of interest (Additional file 3). For the auditory stimulus, the *fmr1*^*−/−*^ brain had more broadly distributed responses (Fig. 2g), especially in the cerebellum, hindbrain, and octavolateralis nucleus (homologous to the cochlear nucleus in mammals). There was also a trend towards more auditory responsive ROIs in several brain regions (Fig. 2h). In most regions, the strengths (Fig. 2i; Additional file 3) and *r*^*2*^ values (Fig. 2j; Additional file 3) of responses were similar, but ROIs in the octavolateralis nucleus were, although more numerous, weaker and noisier in *fmr1*^*−/−*^ larvae. In summary, network-level alterations in visual motion processing were not detected in *fmr1*^*−/−*^ animals. In contrast, auditory processing appeared substantially more abundant and dispersed in *fmr1*^*−/−*^ animals, but these trends did not consistently reach significance. It may be that the use of a single strong auditory stimulus (white noise at 84 dB) was insufficient for revealing nuanced alterations within the network.

**Fig. 3:**
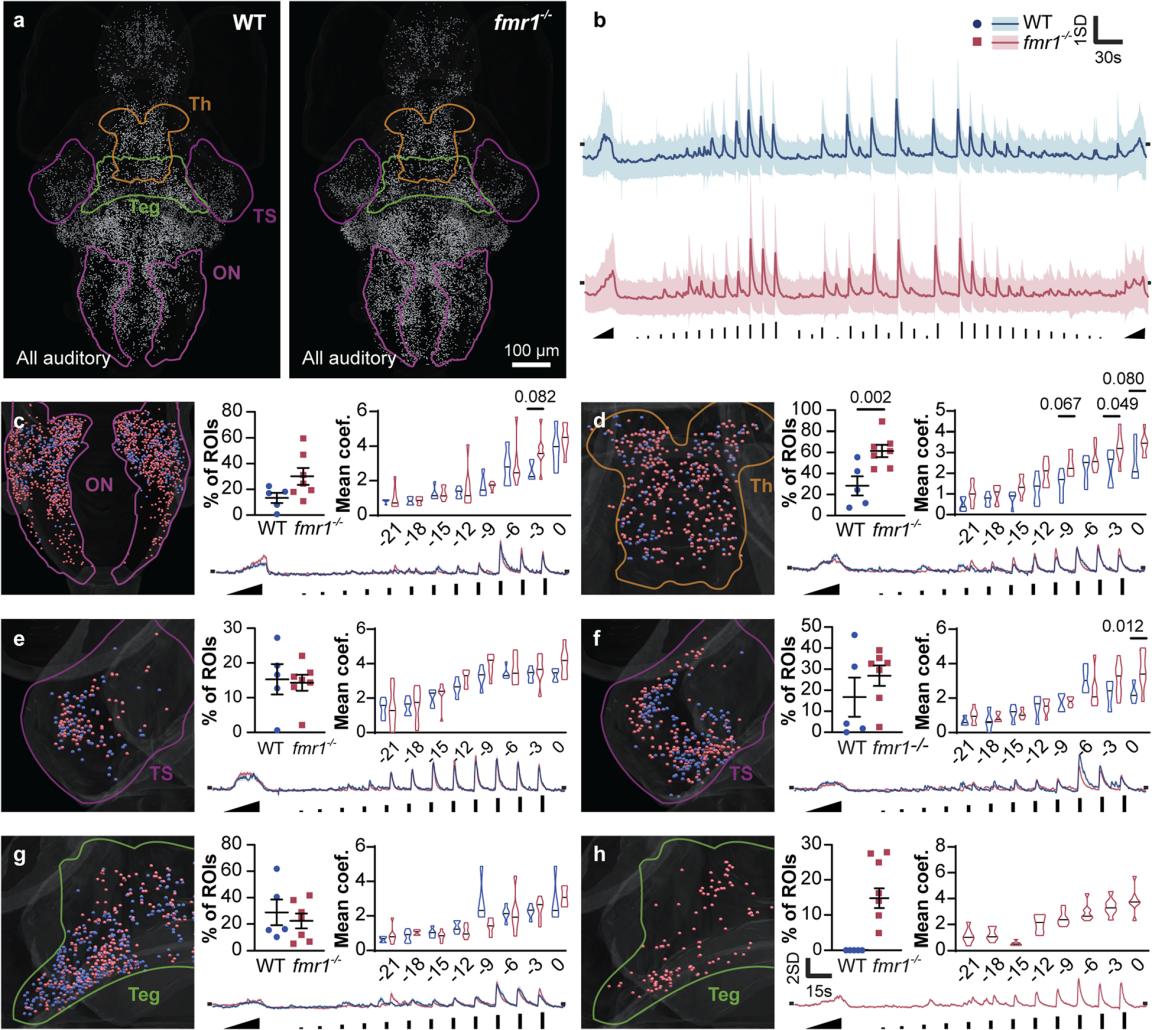
Regional responses to a complex auditory stimulus train. Auditory responsive ROIs (**a**) in WT and *fmr1*^*−/−*^ larvae (equivalent to five larvae). *Z*-scored activity trace (**b**) of all responsive ROIs (mean ± SD) with stimulus timing and amplitude represented (bottom). For each of six functional clusters across four brain regions (**c**–**h**), the distribution of responsive ROIs (left), percent of all ROIs belonging to the cluster, mean response strength to each of eight stimulus amplitudes (top right), and mean *z*-scored activity trace during the first amplitude ramp and first twelve discrete amplitudes (bottom right). Spheres (**c**–**h**, left) are color coded to genotype. Amplitudes are represented in decibels (dB) from full volume. ON, octavolateralis nucleus; Teg, tegmentum; TS, torus semicircularis; Th, thalamus. *P* values ≤ 0.1 are shown

To delve deeper into the auditory phenotype, we designed an auditory-only stimulus train comprising an ascending amplitude ramp, played twice, and twelve amplitudes of a brief auditory stimulus, played three times each. For simplicity, we will refer to our previously-used auditory tone (Fig. 2) as 0dB, with quieter stimuli expressed in negative dB relative to this. The overall distribution of responsive neurons (Fig. 3a) was similar to that observed with our simple auditory stimulus (Fig. 2g), with a large number of broadly distributed auditory ROIs. The mean *z*-scored fluorescent traces of brain-wide auditory ROIs were similar in WT and *fmr1*^*−/−*^ larvae, with a few examples of responses to weak stimuli in *fmr1*^*−/−*^ larvae that were absent from WT larvae (Fig. 3b).

We next performed an in-depth analysis of auditory responses in the auditory sensitivity dataset, covering the same ten brain regions analyzed in the multisensory dataset (Fig. 2). Unsurprisingly, many of the brain regions that displayed altered auditory responses in *fmr1*^*−/−*^ animals are known to play roles in auditory [36, 37] and vestibular processing [38], both of which are reliant on hair cell receptors and the vestibulocochlear nerve. We applied *k*-means clustering on the time series, with the city block metric, to identify functionally distinct categories (clusters) of ROIs with consistent and characteristic responses to our auditory stimuli. For each brain region and cluster, we measured the proportion of all ROIs that belonged to the relevant cluster, compared their mean *z*-scored fluorescent traces, and quantified their average response strengths at each auditory amplitude (Additional file 4). Four brain regions showed trends or significant differences in *fmr1*^*−/−*^ versus WT (Fig. 3c–h), with each cluster in each region and genotype represented by at least 80% of the larvae (assuring that the observed effects were not artifacts from individual animals). Beginning with the octavolateralis nucleus, the first brain region to receive auditory input from the vestibulocochlear nerve (cranial nerve VIII) [39], we identified a single functional cluster with substantially more ROIs in *fmr1*^*−/−*^ larvae. This difference was the result, at least in part, of auditory responses extending more caudally into the octavolateralis nucleus (Figs. 2g and 3a, c). In the thalamus (Fig. 3d), there were significantly more auditory-responsive neurons in *fmr1*^*−/−*^ larvae, combined with an increase in the response strength of these ROIs across a range of amplitudes. Two separate functional clusters emerged in the torus semicircularis. The first cluster (Fig. 3e) was incrementally sensitive to a wide range of amplitudes and showed no significant differences in the proportions, response strengths, or response traces between genotypes. The second, less sensitive cluster of the torus semicircularis (Fig. 3f) responded to stronger amplitudes in *fmr1*^*−/−*^ larvae. These ROIs were more numerous and had significantly elevated response strengths at higher amplitudes. A cluster with similar response characteristics was present in the tegmentum (Fig. 3i), where there were no pronounced differences between genotypes. A second tegmental cluster was identified exclusively in *fmr1*^*−/−*^ animals (Fig. 3h**)**, with response strengths that better reflected the stimulus intensity across a range of amplitudes. Thus, four brain regions in *fmr1*^*−/−*^ animals showed functional differences in intra-regional auditory processing.

**Fig. 4:**
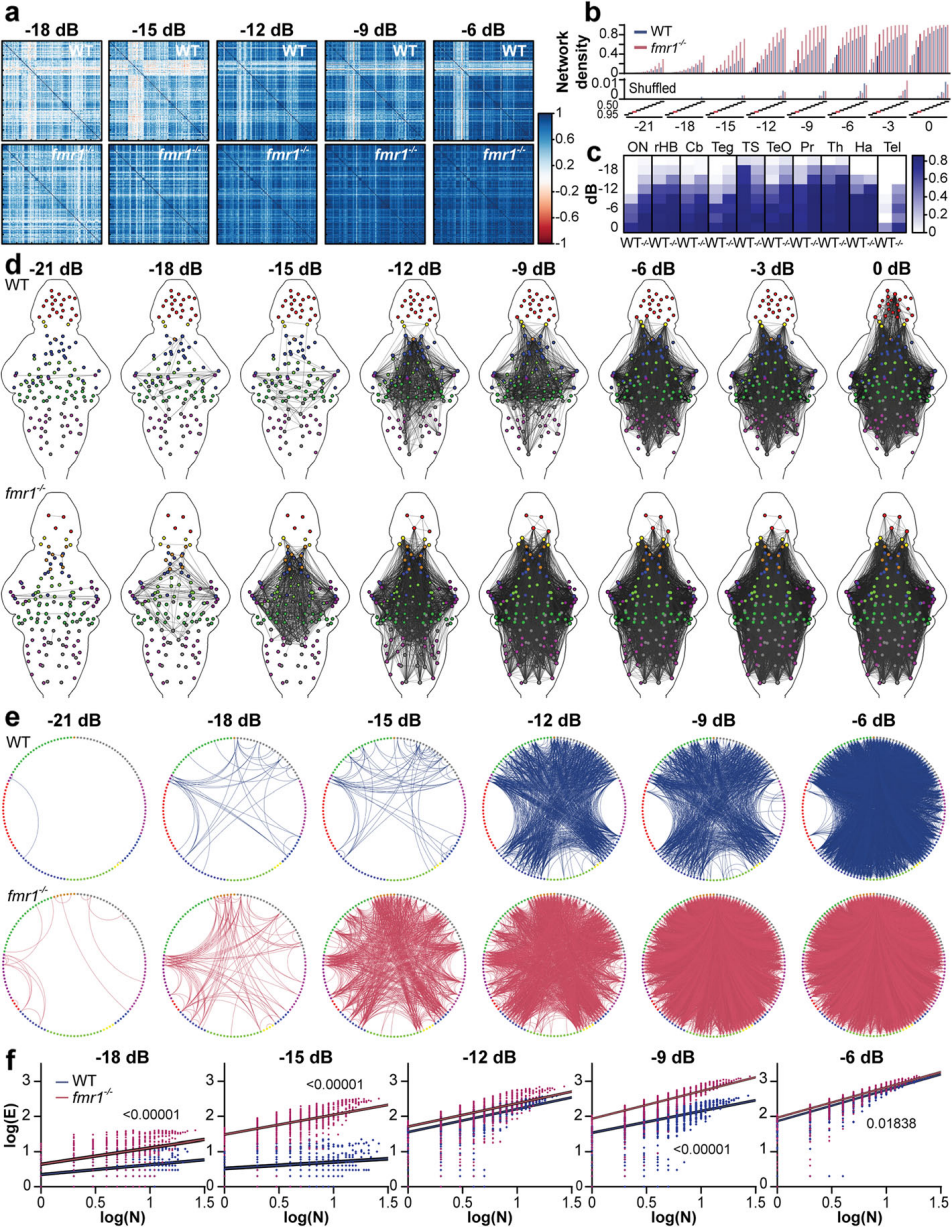
Functional brain-wide auditory networks in WT and *fmr1*^*−/−*^ larvae. Correlation matrices (**a**) showing pairwise correlation strength across all pairs of nodes in WT (top) and *fmr1*^*−/−*^ (bottom) larvae. Amplitudes are annotated as decibels from full volume (dB). Network density (**b**) of auditory sensitivity (top) and time-shuffled dataset (bottom) as a function of correlation coefficient thresholds and select. The 0.85 correlation coefficient threshold (red dash) was selected for subsequent analyses. The mean participation coefficient (**c**) for each region across eight amplitudes. Brain-wide auditory networks (**d**) showing edges exceeding a correlation coefficient of 0.85. Node color indicates brain region: octavolateralis nucleus (ON), magenta; cerebellum (Cb), dark green; hindbrain without the Cb and ON (rHB), gray; tegmentum (teg), light green; torus semicircularis (TS), dark magenta; optic tectum (TeO), blue; pretectum (Pr), light blue; thalamus (Th), orange; habenulae (Ha), yellow; telencephalon (Tel), red. Circle plots (**e**) of strongly correlated edges between nodes located in different brain regions (colored as **d**) for different sound amplitudes in WT and *fmr1*^*−/−*^. Log plots (**f**) of the number of nodes within a box (N) versus the number of edges crossing the box boundary (E) used to calculate the *Rent exponent*. Each dot represents an individual randomly placed and sized box (*n* = 5000), and the lines are robust linear regressions ± SD. *P* values ≤ 0.1 are shown

We next used graph theory to examine the impact of the *fmr1* mutation on inter-regional, or brain-wide, auditory networks. For the WT and *fmr1*^*−/−*^ datasets, we generated sets of 132 and 134 nodes, respectively, which represented the functional units of audition across the ten brain regions of interest. The flow of information was modeled by calculating the correlation coefficient across all pairs of nodes. Matrices representing correlations between these pairs showed stronger associations in *fmr1*^*−/−*^ animals across all amplitudes tested (Fig. 4a). In *fmr1*^*−/−*^ larvae, we found enhanced density, which measures the portion of each node’s possible edges that were strongly correlated across the whole network, a result that was consistent across all amplitudes and correlation thresholds (Fig. 4b). The network densities of the responsive nodes, when compared to time-shuffled data (Fig. 4b; Additional file 5), were well above chance. Participation, a measure of each brain region’s correlation with other brain regions’ nodes, was increased in *fmr1*^*−/−*^ larvae for all regions tested except the torus semicircularis (Fig. 4c). These results show greater inter-regional functional correlations for auditory information in the *fmr1*^*−/−*^ brain. We next mapped all the nodes back to a reference brain (nodes were bounded by brain regions) and represented strong pairwise correlations as edges. These graphs showed stronger inter-regional correlations in the *fmr1*^*−/−*^ brain that were consistent across a range of stimulus amplitudes (Fig. 4d).

To identify the brain regions more strongly correlated in *fmr1*^*−/−*^ animals, we used graph theory to organize the auditory-responsive nodes by region and graphed the strongly correlated edges at different stimulus amplitudes (Fig. 4e). This analysis revealed more functionally correlated regions at lower amplitudes in *fmr1*^*−/−*^ animals, with a greater number of brain regions engaging earlier in the range of amplitudes. In WT larvae, at − 21 dB, two edges formed between the left and right hemispheres of the torus semicircularis, and the torus semicircularis and optic tectum. Increases in amplitude led to numerous interactions between the torus semicircularis and the tectum, hindbrain, and pretectum, in addition to edges between the thalamus and pretectum. As the amplitude increased further, the cerebellum engaged, and by − 9 dB, all ten brain regions of interest were engaged. In contrast, at − 21 dB, *fmr1*^*−/−*^ larvae had fourteen edges form between predominantly midbrain structures, including the two hemispheres of the torus semicircularis, the torus semicircularis and tectum. By − 18 dB (as opposed to − 9 dB in WT), nearly all brain regions were engaged in *fmr1*^*−/−*^ larvae. While the patterns of correlations among brain regions were grossly similar between genotypes, WT responses engaged at sound amplitudes between 3 and 6 dB louder (equivalent to a doubling of amplitude) than *fmr1*^*−/−*^ larvae. Overall, this indicates that *fmr1*^*−/−*^ larvae have auditory networks that engage at lower sound amplitudes than their WT counterparts.

Next we employed the analytical method of Rentian scaling to explore the topological organization of these networks. Rent’s rule defines the scaling relationship between the number of edges crossing a module (E) and the number of connected nodes within a module (N) [40, 41]. This relationship scales according to a power-law in a log-log scale, where the *Rent exponent* describes proportionality. To estimate the *Rent exponent*, we placed 5000 randomly sized boxes over our network and counted the number of nodes (N) and edges (E) crossing the boundaries of the boxes. We then used a robust linear regression between log(N) and log(E) to limit the effects of outliers (Fig. 4f). Subsets of nodes that interact more strongly with each other than the rest of the network are more modular and have a higher *Rent exponent*, and hence, Rentian scaling can be used to uncover the hierarchical modularity of a system and provide insight into inter-module communication. We found that the *fmr1*^*−/−*^ network had a significantly higher exponent than the WT network at lower sound intensities (− 18, − 15, and − 9 dB, although not at − 12 dB) and that edge numbers between boxes increased at a faster rate across amplitudes in *fmr1*^*−/−*^ (Additional file 6). This implies that *fmr1*^*−/−*^ larvae have more inter-module connectivity during auditory processing. These findings support our previous observations of greater participation between brain regions (with the exception of the torus semicircularis) in *fmr1*^*−/−*^ larvae (Fig. 4c). In addition to revealing insights into the hierarchical modularity of a system, Rentian scaling also measures the cost-efficiency of a network. The greater the *Rent exponent*, the more random, long range, and complex the wiring of the system, at the cost of a more economical design. On the other hand, more economy in design (a smaller *Rent exponent*) reduces the metabolic costs of cellular growth and organization between different brain regions. The right balance needs to be achieved between wiring minimization and optimal complexity in biological systems. The consistently greater *Rent exponent* as sound amplitude increases in *fmr1*^*−/−*^ animals suggests that these auditory networks are more complex in wiring and perhaps have lower efficiency.

Finally, we addressed two alternative explanations for increased brain-wide correlations in *fmr1*^*−/−*^ animals*.* The first possible explanation is that the enhanced network activity in response to auditory stimuli stems from more frequent body movements, which would result in highly correlated artifacts across the network. Given that hyperactivity is a commonly reported feature of *fmr1*^−/−^ animal models, including in *fmr1*^*hu2787*^ zebrafish larvae [42], hyperactivity could account for the increased network correlations in *fmr1*^−/−^ larvae. To address this possible confound, we measured head displacement in the *Y*-axis during the brain-wide calcium imaging time series for the auditory sensitivity experiments. We estimated motions caused by startles and swim attempts, and found no significant differences in baseline or stimulus-induced movements in *fmr1*^*−/−*^ larvae (Additional file 7). Indeed, baseline movements were slightly higher in WT larvae. Therefore, differences in baseline motion during imaging did not account for the increased network-wide correlations observed in *fmr1*^−/−^ larvae during auditory stimulation. A second possible explanation for the stronger inter-regional, or brain-wide, correlations in *fmr1*^*−/−*^ larvae to sound is that responses were more plentiful. To assure that response strengths, rather than more plentiful responses, influenced network activity over the range of amplitudes, we generated a set of 49 nodes (identical between WT and *fmr1*^*−/−*^ larvae) that represented the functional units of audition across the ten brain regions. We observed more correlated edges in *fmr1*^*−/−*^ larvae at lower sound amplitudes in the node-matched graphs (Additional file 8), equivalent to 3 dB shift in the sensitivities of *fmr1*^*−/−*^ larvae versus WT, and observed similar effects when sorting the nodes by their brain regions (Additional file 8). This supports our conclusion that the graph density changes in *fmr1*^*−/−*^ animals are driven by stronger (and not more plentiful) neural responses.

**Fig. 5:**
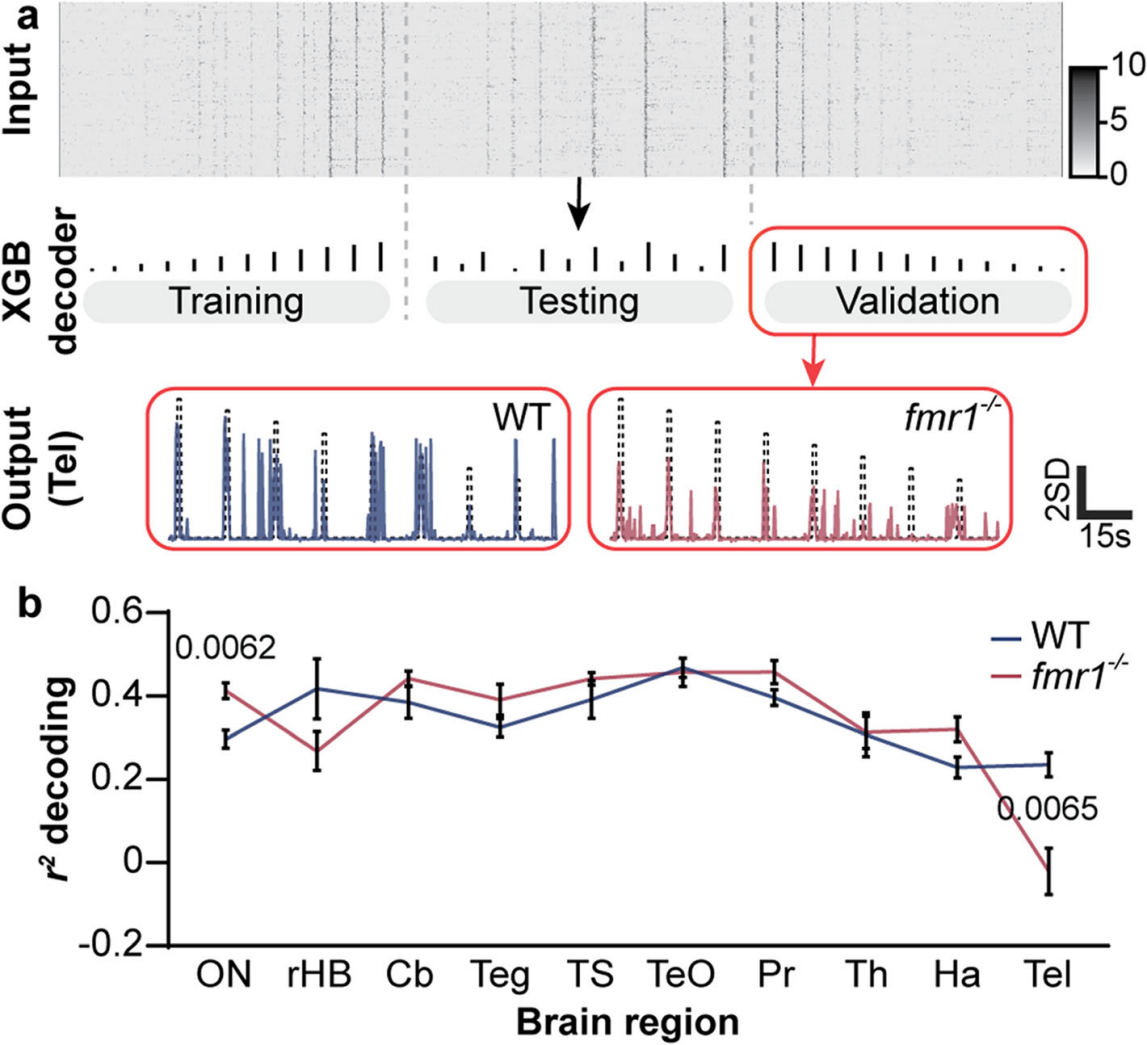
Population decoding of sound amplitude in different WT and *fmr1*^*−/−*^ brain regions. Workflow (**a**) of population decoding analysis. For each brain region of interest, the neuronal activity in ROIs for each genotype (raster plot, top) was randomly split into 10 cross-validation subsets. “Ascending” auditory stimuli were fed to train the Xtreme Gradient Boosting (XGB) decoder, the “quasi-random” auditory stimuli were used to test the decoder, and the “descending” auditory stimuli to validate the predictions. The output of the decoder analysis for the telencephalon (tel) is shown (bottom) as a time trace of the stimulus features (black dotted line) versus predicted responses (colored solid line). Mean *r*^*2*^ (**b**) of auditory clusters decoding amplitude intensity in WT and *fmr1*^*−/−*^ larvae for brain regions of interest. Note brain regions are depicted in approximate order of the ascending sensory pathway. ON, octavolateralis nucleus; rHB, remaining hindbrain (without the cerebellum (Cb) and ON); Teg, tegmentum; TS, torus semicircularis; TeO, optic tectum; Pr, pretectum; Th, thalamus; Ha, habenulae; Tel, telencephalon. Corrected *P* values ≤ 0.01 are shown

Besides stronger brain-wide correlations to auditory stimulation, our data also hint at alterations to the way sensory signals are processed along the ascending auditory pathway (Fig. 3c–i). To better understand how this transmission is altered in *fmr1*^*−/−*^ animals, we used a form of pattern classification called population decoding. Population decoding analysis uses patterns of neuronal activity to predict the experimental conditions across trials [43]. In this case, we used the patterns of neuronal activity within different brain regions to make predictions about which sound amplitudes were presented. We measured the accuracy of this process by calculating the *r*^*2*^ value between prediction and the actual amplitudes for each brain region. This enabled us to assess how well sound amplitude information was encoded, with higher *r*^*2*^ values representing a better representation (Fig. 5a). Decoder analysis revealed that the octavolateralis nucleus, early in the auditory pathway, was significantly better at decoding sound amplitude in *fmr1*^*−/−*^ larvae, which suggests that information about the amplitude of auditory stimuli is encoded more directly in the mutant octavolateralis nucleus (Fig. 5b). In the telencephalon, the encoding of sound amplitude was significantly worse in *fmr1*^*−/−*^ than WT animals (Fig. 5b), suggesting that the later stages of auditory processing in mutants contains less information about the salient features of auditory stimuli. In regions that lie between the octavolateralis nucleus and the telencephalon, or that are parts of other streams of auditory processing, coding appeared normal.

## Discussion

### Alterations in auditory responses for FXS

In this study, we mapped visual and auditory information processing spanning individual neurons, local populations, and brain-wide networks. Across these scales, we did not detect differences in visual processing. However, we identified several major differences in auditory processing in our FXS zebrafish model compared to WT. First, *fmr1*^*−/−*^ larvae had more auditory responsive neurons in the primary auditory regions (in the hindbrain and octavolateralis nucleus) that were more caudally distributed. The thalamus also contained significantly more auditory-responsive neurons. Second, *fmr1*^*−/−*^ larvae had stronger responses to sound in at least three brain regions in the ascending auditory pathway. The thalamus showed stronger responses across a range of amplitudes, while a “less sensitive” cluster of neurons in the torus semicircularis and a unique cluster in the tegmentum showed enhanced response strengths. Third, *fmr1*^*−/−*^ larvae were more sensitive to softer sound amplitudes. Graph theory uncovered higher correlations among virtually all auditory responsive brain regions in *fmr1*^*−/−*^ larvae, from early through to late regions along the auditory processing pathway, in addition to a 3–6-dB shift in the network responsiveness of *fmr1*^*−/−*^ larvae. The 3–6-dB shift suggests that *fmr1*^*−/−*^ animals encode sound intensities at roughly half the amplitude of those encoded by WT larvae. Furthermore, graph theory and Rentian scaling revealed greater participation of virtually all brain regions across all amplitudes, and decoding analysis indicated that the *fmr1*^*−/−*^ octavolateralis nucleus was significantly better at encoding sound amplitude. These data point towards increased sensitivity to auditory stimuli in *fmr1*^*−/−*^ animals that result from perturbations in how sensory information is processed by brain-wide networks. Behavioral auditory hypersensitivity is a characteristic of people with FXS [44], as are increased neural responses to auditory stimuli [9–11].

Alterations in the distributions and response profiles of neurons within brain regions, combined with the correlations between the neurons’ representative nodes across the brain, also revealed fundamental differences in inter-regional, or brain-wide, network properties for the *fmr1*^*−/−*^ auditory phenotype. During auditory processing in zebrafish, information from the vestibulocochlear nerve is first received by the octavolateralis nucleus (homologous to cochlear nuclei) and mediodorsal hindbrain (presumed to develop into the secondary octaval population [39]), which project to the torus semicircularis (homologous to the inferior colliculus) *en route* to the thalamus [36, 37]. The thalamus is reciprocally connected to the dorsomedial telencephalon (homologous to the mammalian amygdala [37]). The torus semicircularis also relays auditory information to the deeper layers of the tectal neuropil (homologous to the superior colliculus [11]) and tegmental nuclei, which form a part of a descending inhibitory reflex circuit with the hindbrain and spine [45]. We identified atypical interactions across the entire auditory processing network in *fmr1*^*−/−*^ larvae, including brain regions homologous to the cochlear nuclei (octavolateralis nucleus in zebrafish), the superior olivary complex (likely the mediodorsal hindbrain in zebrafish larvae), the midbrain inferior colliculus (torus semicircularis in zebrafish), and thalamus. Our findings support clinical observations that FXS fundamentally alters sensory information processing at multiple stages along the ascending pathway [46]. Enhanced and possibly prolonged volume-dependent activity has also been identified in the medial nucleus of the trapezoid body of the superior olivary complex in young *Fmr1* knockout mice [47], which would predict increased synchrony or excitability in the mediodorsal hindbrain of zebrafish.

### The prominent role of the thalamus in sensory processing for FXS

Hyper-connectivity of the thalamus has consistently been reported in ASD [21, 48, 49]. For instance, adolescents with ASD present structural hyper-connectivity between the thalamus and motor/somatosensory cortices [50]. Thalamic and cortical connectivity are also increased [49], and young adults on the autism spectrum have increased functional connectivity between the thalamus and primary sensory cortices that are associated with autistic train severity [48]. The thalamus, often referred to as a “sensory gate”, indirectly receives information from sensory receptors and projects to targeted regions within the cortex. Hyperactivity in the thalamus, likely stemming from impairments in the filtering of sensory information [22], could therefore override the higher-order cognitive processes required to mount appropriate behavioral responses [48].

We identified striking changes in sub-cortical functional connectivity during audition in *fmr1*^*−/−*^ larvae. Our findings are notable because human and mammalian studies emphasize functional connectivity within the cortex. Using zebrafish, we traced the flow of sensory information entering the primary processing areas and then passing through ascending and descending pathways. We identified four sub-cortical brain regions conserved between fish and humans with enhanced auditory responses in *fmr1* mutants. We identified a greater number of responses in the hindbrain and octavolateralis nucleus and higher strengths of responses or *r*^*2*^ values in the tegmentum, torus semicircularis, and thalamus. These results suggest that less filtering takes place at the early stages of the ascending auditory pathway and that these effects persist into later stages of sensory processing. These conclusions were supported by population decoding analysis. The two brain structures with the most altered response profiles were the midbrain and forebrain structures of the tegmentum and thalamus. We also uncovered dramatic increases in correlations between the thalamus and various brain regions, which arose from the combined effects of more plentiful and stronger neuronal responses with stronger links to other brain regions. This has recently been shown in a variety of large-scale multi-site studies in ASD showing hyper-connectivity driven by the thalamus [48, 51–53]. Our analyses therefore suggest that impairments in sensory filtering mechanisms of *fmr1*^*−/−*^ animals stem from thalamic hyperactivity and functional hyper-connectivity.

### Network hyper-connectivity in *fmr1-*mutant zebrafish with relevance to FXS

Functional connectivity studies have provided useful insights into the neurobiological basis of ASD. For instance, the heightened functioning of sensory systems in individuals with ASD has been related to increased local connectivity in occipitotemporal regions, while reduced local connectivity in frontal regions has been linked with disruptive social behaviors [54]. Discrepancies still exist within the literature on whether hypo- or hyper-connectivity contributes to FXS and ASD. In this study, we have addressed these questions at cellular resolution, which has enabled us to identify the strengths of relationships between neurons’ activity patterns within and across brain regions.

Using Rentian scaling and population decoding, we show that the *fmr1*^*−/−*^ network during auditory processing is more complex and responsive (hence more sensitive to quieter stimuli), likely at the cost of a less economically wired circuitry. These observations support the model of functional hyper-connectivity in FXS during development. These findings also support our conclusions that sensory information floods the primary processing regions of the *fmr1*^*−/−*^ brain and that the early ascending auditory pathway inadequately filters the transmission of this information, leading to less efficient coding in some later parts of the auditory pathway. In contrast to our findings, adult *Fmr1*^*−/y*^ mice have reduced functional and structural inter-regional connectivity between the primary visual cortex and neocortex [55]. These discrepancies may result from differences in the sensory modalities, developmental stages, or brain regions analyzed in the studies. There are mixed reports of hyper- and hypo-connectivity in ASD, but most of the studies have insufficient statistical power. A recent large-scale and age-matched meta-analysis of human neuroimaging data convincingly showed significantly more hyper-connectivity in the thalamocortical circuits that failed to decrease with age in ASD [53], as would happen in the typically developing central nervous system. Other large-scale multi-site studies also support hyper-connectivity driven by the thalamus as a general feature of ASD [48, 51, 52]. Thus, based on the results presented here and previous work, the thalamus of *fmr1*^*−/−*^ animals appears to play an important role in the increased correlations and functional hyper-connectivity found in FXS and, perhaps more broadly, ASD.

Brain-wide cellular-resolution analysis, as performed here, is comprehensive. In principle, it reveals all sensory-responsive neurons and allows for differences between genotypes to be described and explored. There are also important limitations to this approach. While we have described correlation strengths between different types of responsive neuron within and between brain regions, we can only speculate about how our observed networks are actually wired. Our observations do not mean that our correlated cell types are directly physically or functionally connected and do not provide insights into the mechanisms by which these correlations are strengthened in *fmr1* mutants. Our brain-wide mapping of these responses should, more appropriately, be viewed as a departure point for more targeted future studies into the morphologies and connectivity of the neurons we describe, the sensory information that flows through these connections, and the specific anatomical or physiological alterations that allow this information to flow more freely and with less filtering in animals bearing mutations in the *fmr1* gene.

## Conclusions

Although auditory hypersensitivity is well documented in FXS and various animal models of the syndrome, we provide a deeper understanding of the underlying network-level changes. We pinpointed alterations in the abundance of auditory responses and their strengths in four sub-cortical brain regions: the octavolateralis nucleus (homologous to the cochlear nucleus), thalamus, torus semicircularis (homologous to the superior colliculus), and tegmentum. We also identified structures with stronger decoding of sound amplitude in *fmr1*^*−/−*^ larvae and telencephalic neurons where this coding was weaker. Furthermore, based on the brain-wide functional connectivity networks of the *fmr1*^*−/−*^ larvae, we demonstrate increased sensitivity to sound intensity by a shift of 3–6 dB. These findings suggest that auditory information floods the primary processing regions of the *fmr1*^*−/−*^ brain, which may lead to the less efficient coding of relevant sensory information in the telencephalon. Our results also suggest that the thalamus plays a pivotal role in the auditory hypersensitivity phenotype. That is, the combined effects of more plentiful and stronger neuronal responses with stronger links to other brain regions of the *fmr1*^*−/−*^ thalamus may explain the stronger correlations and functional hyper-connectivity found in FXS (and perhaps more broadly ASD). The hyperactivity of the thalamus, likely stemming from impairments in the filtering of sensory information, may override the higher-order cognitive processes of the telencephalon.

## Methods

### Animals

Adult zebrafish (*Danio rerio*) were reared and maintained in a zebrafish housing system (Tecniplast, Buguggiate, Italy) under standard conditions using the rotifer polyculture method for early feeding 5 to 9 dpf. Embryos were reared in embryo medium (1.37 mM NaCl, 53.65 μM KCl, 2.54 μM Na_2_HPO_4_, 4.41 μM KH_2_PO_4_, 0.13 mM CaCl_2_, 0.16 mM MgSO_4_, and 0.43 mM NaHCO_3_ at pH ~ 7.2) at 28.5 °C on a 14-h light to 10-h dark cycle. To generate the experimental cohort, zebrafish carrying the *fmr1*^*hu2787*^ allele (kindly provided by Howard Sirotkin) [56] were bred to zebrafish carrying the *elavl3:H2B-GCaMP6* transgene [32]. The fast variant of GCaMP6, *elavl3:H2B-GCaMP6f*, was used to capture baseline and auditory sensitivity activity. The slow variant *elavl3:H2B-GCaMP6s* was used for multisensory-evoked experiments. Zebrafish mutant transgenic animals were out-crossed for four generations to the *Tupfel long fin nacre* WT strain (Zebrafish International Resource Center, Eugene, Oregon).

We generated the experimental cohort by inter-crossing the fourth generation of zebrafish heterozygous for both *fmr1*^*hu2787*^ and *elavl3:H2B-GCaMP6* to produce clutches with a Mendelian ratio of 1:2:1 (WT: *fmr1*^*+/−*^: *fmr1*^*−/−*^). For microscopy experiments, larvae were pre-screened for GCaMP6 expression under a fluorescence microscope at 3 dpf. Genomic DNA was extracted from 6 dpf larvae at the end of imaging sessions and then preserved for long-term storage. Following quality control and the segmentation or larvae tracking stages of data analyses, genotyping for *fmr1* was performed as previously described [57]. We remained blinded to genotype until the later stages of data analysis to minimize systemic biases introduced during experimentation and quality control.

### Calcium imaging

Larvae at 6 dpf were embedded upright in 1.5% low melting temperature agarose inside of a custom-built imaging chamber [58]. Imaging chambers were composed of a 3D-printed base (24 × 24 mm) with four posts (3 × 3 × 20 mm) raised along the four corners of the platform. The four outward faces of the chambers were fixed with a glass coverslip (20 × 20 mm, 0.13–0.16 mm thick). Individual larvae were mounted onto a raised platform (11 × 11 × 6 mm) within each chamber. The platform was no closer than 3 mm to any of the glass surfaces. Chambers were filled with embryo medium once the surrounding agarose had set.

Visual stimuli were displayed on a 75 × 55 mm LCD generic PnP monitor (1024 × 768 pixels, 85 Hz, 32-bit true color) positioned 35 mm lateral to the larva [59, 60] (Fig. 1a). The monitor was covered by a colored-glass filter (Newport, Irvine, CA) with a cut-on wavelength of 550 nm. Auditory stimuli were played from a 9-mm 1-watt haptic feedback and audio exciter (Dayton, Springboro, OH) fixed to the glass surface of the imaging chamber posterior to the larva. The audio exciter was wired to a 2 × 15-watt miniature amplifier (Dayton, Springboro, OH).

In vivo GCaMP6 imaging was performed using a custom-built light-sheet microscope. As the experimentalists were blinded to genotype, all imaging parameters were the same across genotypes. The microscope has previously been described [38], including the line diffuser used to reduce striping artifacts [35]. For multisensory experiments, to avoid the laser paths streaming directly into the eyes (which would interfere with the perception of visual stimuli), we blocked the side laser plane and restricted the front laser plane to the area between the eyes using a custom-made 5-mm wide adjustable slit. For baseline and auditory sensitivity experiments, we used both the side and front laser planes. The exposure time for all experiments was 10 ms. The captured images were binned 4x, yielding a final image resolution was 640 × 540 pixels at 16-bit in a tagged image file format. For baseline and auditory sensitivity experiments, 25 transverse sections at 10-μm increments were recorded at four brain volumes per second for 10 min. For multisensory experiments, 50 transverse sections at 5-μm increments were sampled at two brain volumes per second for 4 min and 7 s.

### Stimulus trains for calcium imaging

For multisensory experiments, we presented three sensory stimuli to each larva three times in a semi-randomized order. The stimuli had a minimum inter-stimulus interval of 3 s. Following 15 s of acclimatization to laser scanning, the brain was imaged for 40 s at rest and then for 3 min and 12 s over the course of the multisensory stimulus train. We presented visual stimuli at a resolution of 1024 × 768 pixels at 20 frames per second. The two visual stimuli were moving vertical bars (visual flow) and an expanding disk (visual loom). The visual flow stimulus comprised eight bars, 128-pixels in width, moving in a caudal to rostral direction at a speed of 21.3° per second for 4 s. Prior to each visual flow, a 12-s linear dimming from white to black-and-white bars occurred, and similarly, a black-and-white to white 12-s linear brightening occurred after each visual flow stimulus (total stimulus duration 28 s). The visual loom consisted of a black 4-pixel diameter circular disk that exponentially expanded (Simple, Fastest; easing of − 80 designed on Adobe Animate v18.0) to an 812-pixel diameter circular disk over 6 s at a minimum angle of ~ 11° and a maximum angle of ~ 90°. A linear brightening from black to white over a 12-s duration followed each visual loom (total stimulus duration 18 s). The auditory stimulus was created using the professional audio software, Live 10 (Ableton, Berlin, Germany), and was normalized to 0 dB relative to full scale. It comprised 1 s of white noise with a 2-ms rise/fall time. The sound level of white noise at full scale was 84 dB (background noise 40–45 dB) and was measured using a 30–120-dB range digital sound level meter (DOSS, Melbourne, Australia) positioned just above the imaging platform, where the larvae would be. The total length of the multisensory-evoked stimulus train was 4 min.

For auditory sensitivity experiments, we presented two 30-s white noise amplitude ramps (to 0 dB) and twelve discrete amplitudes of white noise with a duration of 1 s at 3-dB intervals. We played each discrete amplitude once in three blocks, with an inter-stimulus interval of 14 s within blocks and an inter-block interval of 30 s. Following 15 s of acclimatization to laser scanning, the brain was imaged for 90 s during which the first amplitude ramp was presented. The first block comprised the twelve volumes with increasing amplitudes. The second block was quasi-randomized (− 21, − 27, − 12, − 33, − 9, − 18, − 6, − 24, 0, − 15, − 30, − 3 dB), and the third block had decreasing amplitudes. The second amplitude ramp was presented at the end of the stimulus train. The total length of the auditory stimulus train was 11 min and 45 s.

### Analysis of calcium activity

We analyzed larvae that met the following four criteria: (1) showed robust responses to the first visual loom and auditory stimuli (6 of 77 excluded); (2) survived to the end of the imaging session (1 of 77 excluded); and (3) contained WT or *fmr1*^*−/−*^ larvae (that is, not *fmr1*^*+/−*^ exclusively) during the imaging session (imaging sessions were between 4 to 12 min; 7 of 77 excluded). We used the same animals for the baseline and auditory sensitivity experiments (*n* = 23) and a separate animal cohort for the multisensory experiments (*n* = 29). We measured baseline activity first, followed by auditory sensitivity.

For larvae passing the inclusion criteria, we separated their four-dimensional imaging stacks (time, *x*, *y*, *z*) into individual time series for every *x*-*y* plane using ImageJ v1.52c (Rueden et al., 2017). Each plane was then motion corrected using the Non-Rigid Motion Correction (NoRMCorre) algorithm [61]. ROIs, and their corresponding calcium traces, were extracted, de-mixed, and denoised using the CaImAn package [61] as previously described [36, 38]. Following segmentation, we ensured that the number of ROIs detected within any of the brain regions of interest was within an order of magnitude of the median (11 of 77 excluded). Once genotypes were provided, we found similar numbers of ROIs corresponding roughly to single neurons were detected per larvae (WT = 16,593 ± 1165 across 5 animals; *fmr1*^*+/−*^ = 17,077 ± 1316 across 10 animals; *fmr1*^*−/−*^ = 17,321 ± 670 across 7 animals (mean ± s.e.m.)) (Additional file 9). For multisensory experiments, similar numbers of ROIs were detected per larvae (WT = 45,015 ± 4177 across 7 animals; *fmr1*^*+/−*^ = 49,768 ± 2141 across 17 animals; *fmr1*^*−/−*^ = 40,123 ± 3137 across 5 (mean ± s.e.m.)) (Additional file 10**)**.

Calcium traces of all animals and all planes were subsequently pooled per genotype and *z*-transformed for further analysis using MATLAB v9.5 (MathWorks, Natick, MA). For baseline activity, we computed the correlation between all pairs of ROIs and used their spatial localization to calculate Euclidean distances. We estimated calcium event rates by detecting the peaks in each trace that were separated by at least 2 s and that increased locally by at least 1 SD above the baseline. For evoked activity, we modeled calcium traces using a multivariate linear regression against regressors (using a typical GCaMP6 response) that corresponded to the timing of the relevant stimulus types [38]. For multisensory experiments, we built three regressors for each of the three presentations of visual flow, visual loom, and auditory stimuli. Likewise, for the auditory sensitivity experiments, we built twelve regressors for each of the discrete sound amplitudes.

### Thresholding and calculating responsiveness

For the multisensory dataset, we defined an ROI as responsive to a particular stimulus if it had a regression coefficient 2 SD above the mean of all regression coefficients and had an *r*^*2*^ value greater than 0.1 (26th percentile) in the WT group. WT thresholds were applied to *fmr1*^*+/−*^ and *fmr1*^*−/−*^ groups. Specifically, the regression coefficient thresholds 2 SD above the mean for visual flow, visual loom, and auditory stimuli were 2.0462, 2.0920, and 2.2894, respectively.

For the auditory sensitivity dataset, we defined an ROI as responsive to a particular stimulus if it had a regression coefficient greater than 0 and had an *r*^*2*^ value greater than 0.05 (80th percentile). Lower thresholds were used for the auditory sensitivity dataset compared to the multisensory dataset because we sought to detect all responses to all auditory stimuli in the sensitivity dataset (including low amplitudes) and to accommodate for a longer stimulus train. Given the complexity of the auditory stimulus train, and the low stringency of the thresholds, non-auditory signals were pooled with the signal. To improve the signal-to-noise ratio without increasing the stringency of the thresholds for the auditory sensitivity dataset (considering that we wanted all auditory categories including weak responses), we excluded time points in individual animals that startled at a particular amplitude, as these generally represent motor responses rather than auditory responses. In the WT group, we removed 50 frames flanking the first − 6 dB amplitude in larva 1, the first − 15 dB amplitude in larva 2, the second 0 dB amplitude in larva 3, and the third − 27 dB amplitude in larva 5. In the *fmr1*^*−/−*^ group, we removed 50 frames flanking the first − 6-dB amplitude in larva 4 and the second − 33-dB amplitude in larva 7 (removing a total of 6 trials among 468 in the experiment).

We subsequently applied *k*-means clustering on the time series to produce five components per genotype for each of the ten brain regions of interest. Five was empirically chosen because it produced clusters that were well represented across the fish population and genotypes. All non-auditory clusters were excluded, and then, the remaining auditory clusters of all animals and all planes were pooled together for each region per genotype, unless a striking difference was observed in the average response traces or spatial localizations. Such striking differences were observed three times, in the tegmentum of both genotypes and in the torus semicircularis of *fmr1*^*−/−*^ larvae. As *k*-means forces all ROIs to belong to a cluster, we removed ROIs with a low correlation to the mean of each cluster (correlation > 0.2) to remove additional noise. The resulting ROIs were classified as auditory responsive in the auditory sensitivity dataset.

To calculate the proportion of ROIs responsive to a particular stimulus, ROI numbers above the regression coefficient and *r*^*2*^ thresholds were normalized to the total number of ROIs detected in the whole brain or relevant brain region. To detect changes in the distribution of regression coefficients, the mean regression coefficients were calculated for ROIs with an *r*^*2*^ above threshold (0.1 or 0.05 depending on the dataset). Similarly, to detect changes in the distribution of *r*^*2*^ values, the mean *r*^*2*^ was calculated for ROIs per larvae with a regression coefficient above threshold (+ 2 SD of the WT or 0 depending on the dataset).

### Registration and visualization of calcium activity

Once the motion corrected individual *x*-*y* planes were complete, we used the three dimensional stacks of all animals included in a particular dataset to build a common template with Advanced Normalization Tools [62]. We then registered the template to the *elavl3-H2BRFP* zebrafish line on the Z-brain atlas [62]. The resulting warps were applied to the centroids of all ROIs for each larva, which were then placed into the 294 brain regions defined by the Z-brain atlas as previously described [63]. We visualized the spatial information and classified activity of each ROI using the Unity Editor (Unity Technologies, San Francisco, CA). Specifically, for the multisensory dataset, we represented ROIs as spheres with their diameter representing their *r*^*2*^ value (1 + *r*^*2*^ × 5 in μm), and color based on their regression coefficient value. For the auditory sensitivity dataset, spot size and color were uniform. The template brain, upon which we overlaid this information, was generated by creating an isosurface mesh over the combined masks of the diencephalon, mesencephalon, rhombencephalon, eyes, and telencephalon from the Z-brain Atlas using ImageVis3D (Scientific Computing and Imaging Institute, Salt Lake City, UT).

### Motion detection during calcium imaging

To identify motion cues during brain-wide calcium imaging, *Y*-axis displacement (pixels) was extracted over time (seconds) from maximum pixel projected hyperstacks of the raw calcium imaging time series. The cv_align stacks plugin from ImageJ was used. The *Y*-displacement values were subsequently filtered using a linear moving average filter (window size of 5) to remove non-linearity caused by any larvae not returning to their original positions following movement. Motion was estimated by calculating the area below the moving-average filtered displacement data using trapezoidal numerical integration.

### Graph theory

We defined the number of nodes in our graph by performing *k*-means clustering on the 3-dimensonal coordinates of all ROIs within each of the ten brain regions per genotype into *k* number of clusters. *k* was chosen as the largest number (starting at 20 + number of ROIs/1000) at which at least 10 ROIs from at least 3 different larvae were retained. This was repeated for each brain region and produced a total of 132 nodes in the WT and 134 in the *fmr1*^*−/−*^ cohort. To generate nodes that were matched across genotypes, *k* was defined as aforementioned, but required at least ten ROIs in at least three different fish from each genotype. We repeated this for each of our ten brain regions to produce 49 nodes in the WT and *fmr1*^*−/−*^ cohort. This approach ensured all ROIs that contributed to each node (for both the unmatched and matched-node graphs) exclusively belonged to one of the ten broadly defined brain regions. We generated the mean *z*-scored fluorescent response from all the ROIs belonging to these nodes across all animals. To determine whether the network effects observed were greater than those expected by chance, we used an Amplitude Adjusted Fourier Transform [64] to generate a temporally shuffled time series which conserves the statistical properties of the original. To avoid shuffling the responses out of the time windows we used to study the different stimuli, we performed shuffling within each time window corresponding to each stimulus for all datasets. The mean responses, of both the shuffled and unshuffled datasets, were used to build correlation matrices for each genotype and binarized with a threshold of 0.85 correlation, from which we built undirected graphs. The Brain Connectivity Toolbox [65] was used to calculate network measurements of the graph from each genotype, such as the density or the participation coefficient between brain regions, as previously described [66]. It was also used to measure topological Rentian scaling using the rentian_scaling_3d function with 5000 boxes on the same binarized correlation matrices as the graphs, with the 3d *z*-scores coordinates of each nodes as inputs. The robust linear regression was computed in Matlab with the defaults settings. Circle plots were produced using the circularGraph toolbox.

### Neural Decoder analysis

We performed decoder analysis using the Neural Decoding toolbox (https://github.com/KordingLab/Neural_Decoding) [43] on the auditory sensitivity dataset. The discrete amplitudes of sound were the experimental features used to decode patterns of activity using the Xtreme Gradient Boosting (XGB) model. For each brain region per genotype, auditory-responsive neurons were randomly divided into 10 subsets. For each of the 10 subsets, the first trial (with the ascending auditory stimulus block) was used to train the XGB model. The XGB model was tested on the second trial (with the quasi-random auditory stimulus block) and then validated on the third presentation (with the descending auditory stimulus block). The *r*^*2*^ was calculated between the predicted features and the actual sound amplitudes.

### Statistical analyses

Significance between two genotypes was tested using unpaired two-tailed *t* tests with the Holm-Sidak (or Sidak for Rentian scaling) method for multiple comparisons. Genotypes showed different variations; thus, *P* values were computed individually (did not assume consistent SD). Significance between three genotypes were tested using an unpaired Kruskal-Wallis test with the Dunn’s multiple comparisons method. Given that data were based on counts (which is positively skewed because they are truncated at zero), the Kruskal-Wallis test was selected because it does not assume that data is sampled from Gaussian distributions. All statistical analyses were performed in Prism v8.0.1 (GraphPad, San Diego, CA).

## Supplementary information

**Additional file 1. **Summary of the statistical analyses used to quantify baseline activity. Brain-wide firing rates and correlation coefficient statistics in WT (*n* = 5), *fmr1*^*+/−*^ (*n* = 10) and *fmr1*^*−/−*^ (*n* = 7) larvae.

**Additional file 2. **Consistency of calcium responses between repeated auditory stimuli. The probability distribution of mean correlations between each ROIs’ responses to repeated stimuli in the auditory sensitivity dataset (mean ± s.e.m.). Auditory responses to stimuli between − 21 and 0 dB from full volume (i.e. 3 repetitions of 8 stimuli) were analyzed in WT (*n* = 5) and *fmr1*^*−/−*^ (*n* = 7) larvae.

**Additional file 3. **Summary of the statistical analyses used to quantify results from the multisensory experiment. Proportion of total, mean regression coefficient, and coefficient of determination (*r*^*2*^) values of ROIs in WT (*n* = 7) and *fmr1*^*−/−*^ (*n* = 5) larvae responding to visual flow, visual loom or auditory stimuli. WB, whole brain; Tel, telencephalon; Ha, habenulae; Th, thalamus; Pr, pretectum; TeO, optic tectum; TS, torus semicircularis; Teg, tegmentum; Cb, cerebellum; rHB, remaining hindbrain (without the Cb and octavolateralis nucleus (ON)).

**Additional file 4. **Summary of the statistical analyses used to quantify auditory sensitivity. *P* values of proportion of total ROIs responsive to all audition (not amplitude specific), and *P* values of mean regression coefficient for brain region-specific clusters of interest above the *responsive threshold* in WT (*n* = 5) versus *fmr1*^*−/−*^ (*n* = 7) larvae at various amplitudes in dB from full volume. Th, thalamus; TS, torus semicircularis; Teg, tegmentum; octavolateralis nucleus, ON.

**Additional file 5. **Correlation matrices and network density measures of the time-shuffled auditory sensitivity dataset. Correlation matrices of the time-shuffled auditory sensitivity dataset showing pairwise correlation strengths across all pairs of nodes in WT (*n* = 5) **(top)** and *fmr1*^*−/−*^ (*n* = 7) **(bottom)** larvae. Amplitudes are annotated as dB from full volume.

**Additional file 6. **Robust linear regression slopes of log(N) versus log(E) of the auditory sensitivity graphs. We applied a robust linear regression to the log(N) versus log(E) plots in Figure 4f, 2-way ANOVA with Sidak correction was used to compare WT (*n* = 5) versus *fmr1*^*−/−*^ (*n* = 7) larvae at various amplitudes in dB from full volume.

**Additional file 7. **Motion during brain-wide calcium imaging. Motion cues were approximated by measuring the total area of Y-axis displacement (in pixels × second) in WT (*n* = 9), *fmr1*^*+/−*^ (*n* = 10) and *fmr1*^*−/−*^ (*n* = 12) larvae over the entire course of the auditory sensitivity stimulus train **(a)** (mean ± s.e.m.), or during the three repetitions of 1 s auditory stimuli at various amplitudes in dB from full volume **(b)** (mean ± SD).

**Additional file 8. **Functional brain-wide auditory networks with matched nodes in WT and *fmr1*^*−/−*^ larvae. Brain-wide auditory networks **(a)** showing edges exceeding a correlation coefficient of 0.85 in nodes matched in WT (*n* = 5) **(top)** and *fmr1*^*−/−*^ (*n* = 7) **(bottom)** larvae. Node color indicates brain region: octavolateralis nucleus (ON), magenta; cerebellum (Cb), dark green; hindbrain without the Cb and ON (rHB), grey; tegmentum (teg), light green; torus semicircularis (TS), dark magenta; optic tectum (TeO), blue; pretectum (Pr), light blue; thalamus (Th), orange; habenulae (Ha), yellow; telencephalon (Tel), red. Circle plots **(b)** showing the locations of genotype-matched nodes (WT, blue; *fmr1*^*−/−*^, red) for strongly correlated edges for various sound amplitudes.

**Additional file 9. **Summary of segmentation for baseline and auditory sensitivity dataset. Total number of ROIs detected per fish for the baseline and auditory sensitivity dataset in WT (*n* = 5) and *fmr1*^*−/−*^ (*n* = 7) larvae. WB, whole brain; Tel, telencephalon; Ha, habenulae; Th, thalamus; Pr, pretectum; TeO, optic tectum; TS, torus semicircularis; Teg, tegmentum; Cb, cerebellum; rHB, remaining hindbrain (without the Cb and medial octavolateralis nucleus (ON)).

**Additional file 10. **Summary of segmentation for multisensory dataset. Total number of ROIs detected per fish for the multisensory dataset in WT (*n* = 7) and *fmr1*^*−/−*^ (*n* = 5) larvae. WB, whole brain; Tel, telencephalon; Ha, habenulae; Th, thalamus; Pr, pretectum; TeO, optic tectum; TS, torus semicircularis; Teg, tegmentum; Cb, cerebellum; rHB, remaining hindbrain (without the Cb and octavolateralis nucleus (ON)).

## Data Availability

The datasets generated during and/or analyzed during the current study are available in the University of Queensland eSpace repository, 10.14264/06dea52 [67], 10.14264/cf6e784 [68], and 10.14264/aa37c6d [69].
