# Supplementary figures and images for: Altered brain-wide auditory networks in a zebrafish model of fragile X syndrome
Source: BMC Biol. 2020 Sep 16;18:125. doi: 10.1186/s12915-020-00857-6 (PMC7493858; doi:10.1186/s12915-020-00857-6)

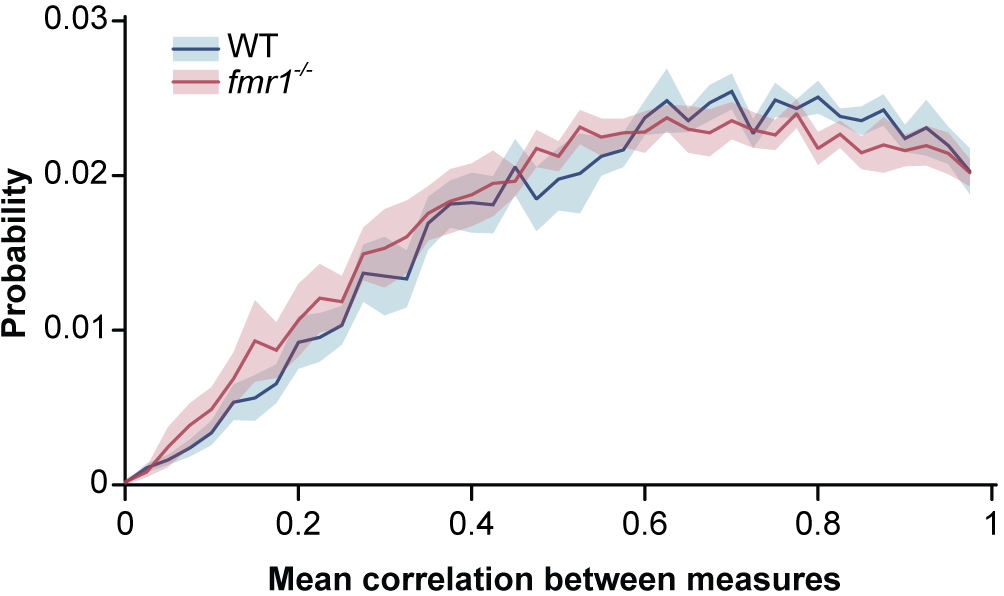

Supplement: Supplementary file 2 — Additional file 2. Consistency of calcium responses between repeated auditory stimuli. The probability distribution of mean correlations between each ROIs’ responses to repeated stimuli in the auditory sensitivity dataset (mean ± s.e.m.). Auditory responses to stimuli between − 21 and 0 dB from full volume (i.e. 3 repetitions of 8 stimuli) were analyzed in WT (n = 5) and fmr1−/− (n = 7) larvae. [file 12915_2020_857_MOESM2_ESM.tif]

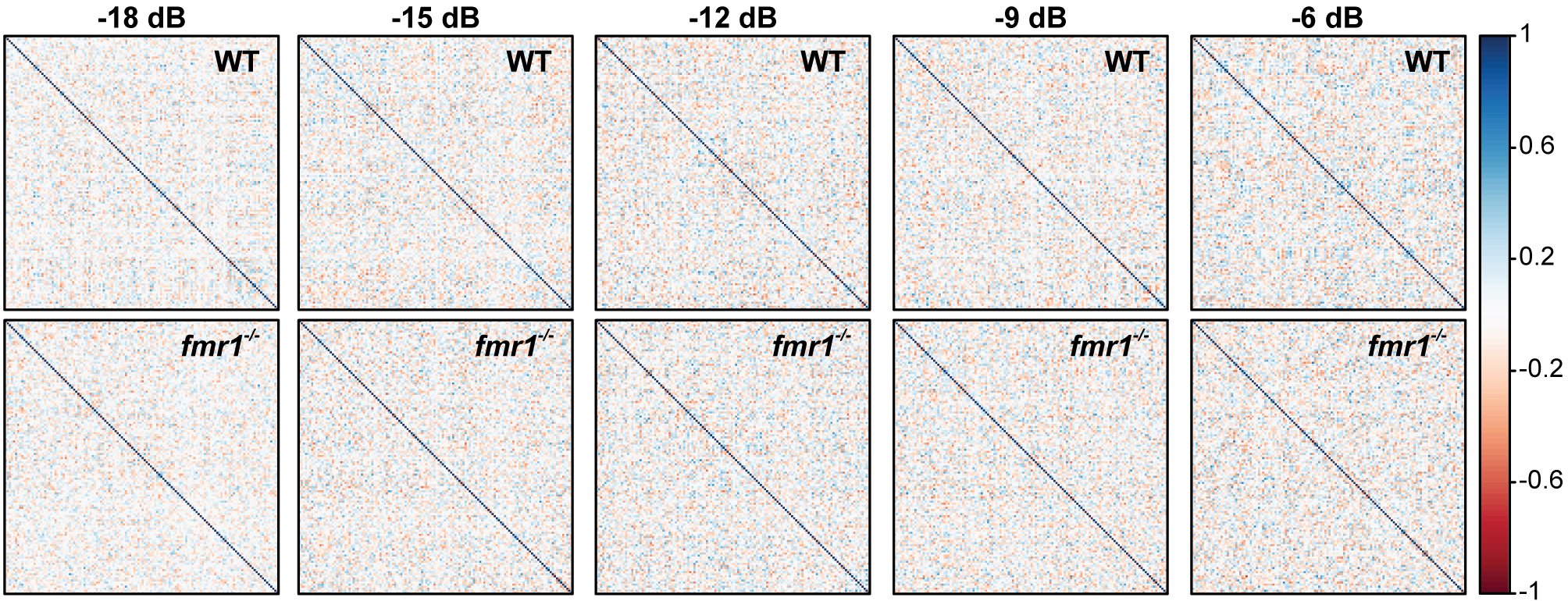

Supplement: Supplementary file 5 — Additional file 5. Correlation matrices and network density measures of the time-shuffled auditory sensitivity dataset. Correlation matrices of the time-shuffled auditory sensitivity dataset showing pairwise correlation strengths across all pairs of nodes in WT (n = 5) (top) and fmr1−/− (n = 7) (bottom) larvae. Amplitudes are annotated as dB from full volume. [file 12915_2020_857_MOESM5_ESM.tif]

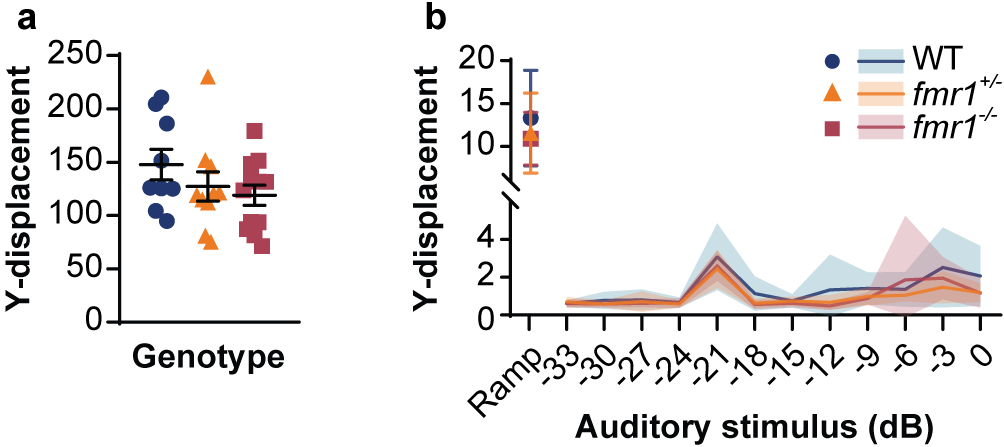

Supplement: Supplementary file 7 — Additional file 7. Motion during brain-wide calcium imaging. Motion cues were approximated by measuring the total area of Y-axis displacement (in pixels × second) in WT (n = 9), fmr1+/− (n = 10) and fmr1−/− (n = 12) larvae over the entire course of the auditory sensitivity stimulus train (a) (mean ± s.e.m.), or during the three repetitions of 1 s auditory stimuli at various amplitudes in dB from full volume (b) (mean ± SD). [file 12915_2020_857_MOESM7_ESM.tif]

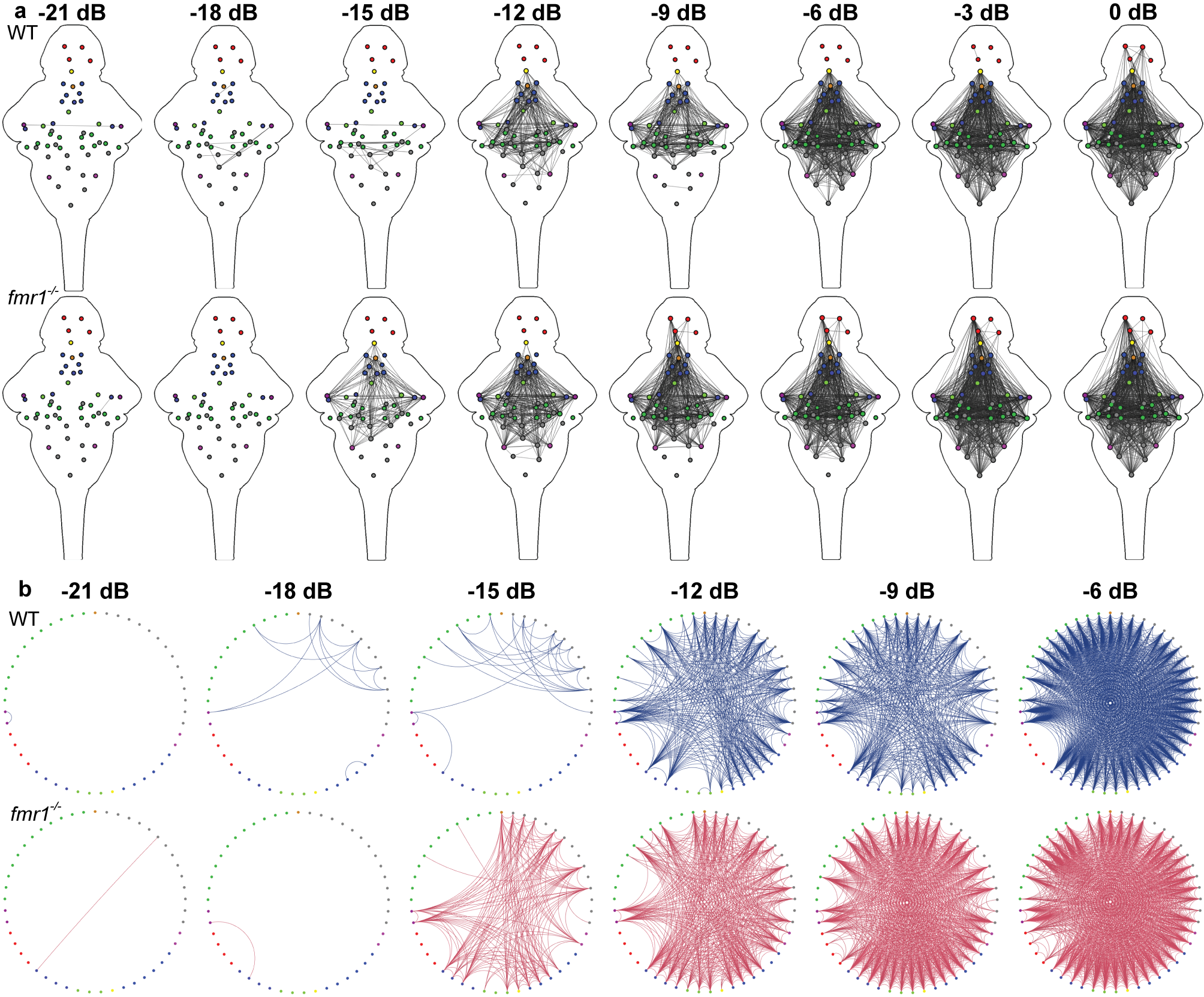

Supplement: Supplementary file 8 — Additional file 8. Functional brain-wide auditory networks with matched nodes in WT and fmr1−/− larvae. Brain-wide auditory networks (a) showing edges exceeding a correlation coefficient of 0.85 in nodes matched in WT (n = 5) (top) and fmr1−/− (n = 7) (bottom) larvae. Node color indicates brain region: octavolateralis nucleus (ON), magenta; cerebellum (Cb), dark green; hindbrain without the Cb and ON (rHB), grey; tegmentum (teg), light green; torus semicircularis (TS), dark magenta; optic tectum (TeO), blue; pretectum (Pr), light blue; thalamus (Th), orange; habenulae (Ha), yellow; telencephalon (Tel), red. Circle plots (b) showing the locations of genotype-matched nodes (WT, blue; fmr1−/−, red) for strongly correlated edges for various sound amplitudes. [file 12915_2020_857_MOESM8_ESM.tif]
